# Phase Morphology of NR/SBR Blends: Effect of Curing Temperature and Curing Time

**DOI:** 10.3390/polym10050510

**Published:** 2018-05-08

**Authors:** Darja Klat, Hossein Ali Karimi-Varzaneh, Jorge Lacayo-Pineda

**Affiliations:** Continental Reifen Deutschland GmbH, Jaedekamp 30, 30419 Hanover, Germany; ali.karimi@conti.de (H.A.K.-V.); jorge.lacayo-pineda.de (J.L.-P.)

**Keywords:** rubber, blend morphology, phase separation, miscibility, atomic force microscopy, transmission electron microscopy, surface free energy

## Abstract

The morphology of natural rubber/styrene–butadiene rubber blends (NR/SBR) was characterized by atomic force microscopy (AFM), with regard to curing temperature and curing time. The changes in blend morphology were directly visualized by AFM which confirmed the results of indirect experiments like differential scanning calorimetry (DSC). Comparing the phase morphologies at different curing temperatures indicated that the domain size of SBR increases with temperature at lower curing temperatures, but domain growing stops at the latest scorch time. This effect is explained by longer scorch times at low curing temperatures which facilitate phase separation, while the short scorch times at higher temperatures meant that the coalescence of SBR phases was hindered by cross-linking between polymer chains.

## 1. Introduction

General purpose elastomers are blended with each other to combine their individual properties in one material. In most cases, natural rubber (NR) is not miscible with synthetic rubbers such as styrene–butadiene rubber (SBR), and therefore, the components of the blend arrange in different domain morphologies [1,2,3,4]. Domain morphology affects the specific aspects of performance in product applications, and therefore, its understanding is crucial.

The resulting morphology can be influenced by many factors like blending ratio, variation in polymer type, and microstructure as well as their polarity, viscosity, and mixing procedure [5]. Miscibility at the molecular level cannot be achieved due to the different surface free energies. An equilibrium phase morphology is reached when the break-down process is in balance with the coalescence of the domains [6]. Molecular weight also influences the domain morphology. While smaller domains are related to similar molecular weight, the domains grow with the increasing difference in molecular weight of the components [7]. The same behavior is observed for the viscosity. The larger domains are formed with a higher viscosity difference between the components [8,9,10,11]. It is widely accepted that with increasing vinyl content in the synthetic rubber the miscibility of NR is improved [12,13,14]. The influence of polymer microstructure on the morphology in NR/BR (butadiene rubber) and NR/SBR have been investigated by several groups, either by direct visualization of the morphology with transmission electron microscopy (TEM) [12], or using dynamical mechanical analysis (DMA) [14].

Besides TEM and DMA, differential scanning calorimetry (DSC) is used as a third standard technique to characterize the miscibility of blends [15,16,17]. Unfortunately, all these methods have their drawbacks. Indeed, TEM provides direct characterization of blend morphology, but the preparation is time consuming and the contrast between the elastomers is quite weak. DSC and DMA investigations have the disadvantage of only characterizing the miscibility of blends indirectly, without giving any information of the real morphology.

Nowadays, AFM is used as an alternative method to conventional approaches, such as TEM, DSC, and DMA, in order to directly visualize the blend morphology [18,19,20]. AFM has the advantage of faster sample preparation. At the same time, AFM offers higher phase contrast between the elastomers without any staining procedure. AFM has an additional advantage, in contrast to TEM, that uncured samples can also be investigated. This fact is especially beneficial for investigating the influence of curing time and temperature.

From earlier works, it is known that blend morphology can be controlled by mixing temperature and mixing time until an equilibrium size of the domain is reached [8]. However, the influence of the curing process on the morphology of rubber blends still requires clarification. Therefore, the objective of this work is to investigate the influence of curing temperature and curing time on morphology in blends cross-linked with sulfur. The study is performed on two different NR/SBR blends. The blends only differ in the vinyl content of SBR. Indeed, Nelson et al. [11] observed changes in the domain size after heat treatment in blends of ethylene–propylene–diene and BR without cross-links.

The results provided in this work indicate that curing temperature and curing time could influence the final phase morphology of NR/SBR blends depending on SBR vinyl content. This finding is important for controlling the blend morphology to achieve a performance target in the final product application. 

## 2. Materials and Methods

### 2.1. Materials 

In this work, NR was blended in a ratio of 70:30 with two different types of SBR. While the styrene content of the two SBR types was the same (24 wt %), the vinyl content differed in both elastomers. One SBR type has a vinyl content of 34.2 wt %, and is called SBR–LV, while the other is referred to as SBR–HV and has a vinyl concentration of 67.2 wt %. Further characteristics of the rubber used in this investigation are summarized in Table 1.

### 2.2. Compounding and Curing

The composition of the compounds is given in Table 2. The compounds were mixed in a 2L-internal mixer (Harburg-Freudenberger, Hamburg, Germany) in a two-step mixing process. In the first step, all ingredients (except in the vulcanization system) were mixed for 3 min at a temperature of approximately 135 °C with a rotor speed of 50 rpm (rounds per minute). In the second step, the vulcanization system was added and the compound was finalized by mixing for 2.5 min at 100 °C with a rotor speed of 30 rpm.

The rheological behavior at constant curing temperatures of 140 and 160 °C was determined with a RPA elite (TA Instruments, New Castle, DE, USA). The frequency and amplitude were 1.67 Hz and 7%, respectively. The temperatures were selected for practical relevance. The lower temperature of 140 °C was critical because the processing time was generally too long for an efficient industrial application, while the higher temperature of 160 °C was also critical because higher values might negatively influence the key physical parameters in a real product [21].

**Figure 1:**
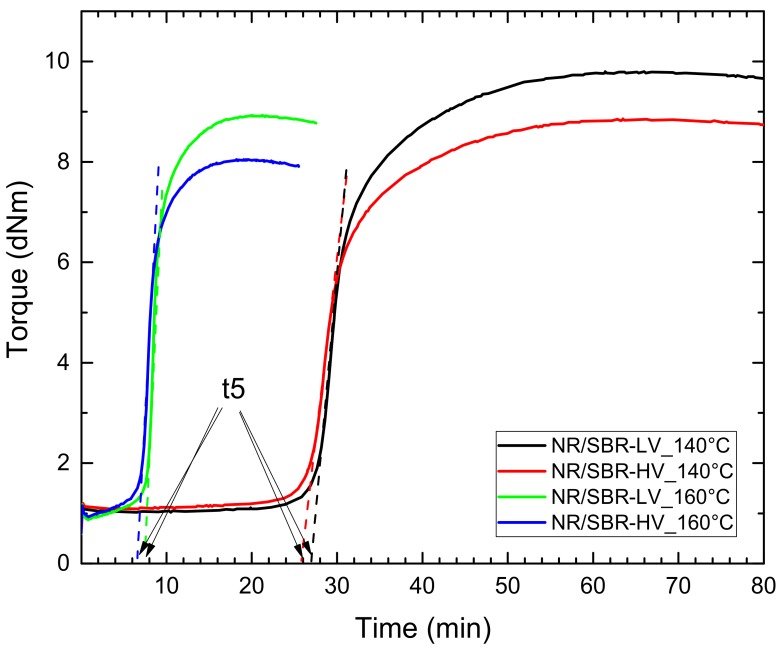
Curing curves (solid lines) of NR/SBR–LV and NR/SBR–HV blends at curing temperatures of 140 and 160 °C. The dashed lines are linear fitting to the curing curves in the region with the steepest slope, and show the approach to define the onset point (t5).

Figure 1 shows the curing curves of the blends at the curing temperatures mentioned above. It is obvious that at the lower curing temperature the scorch time (time until the onset of vulcanization) increased.

The morphology of the blends at the same states of curing was compared: The onset point of curing, defined as the time of 5 percent conversion (t5), was determined by linear fitting of the curing curve in the region with the steepest slope, and extrapolation of the line to zero torque (see Figure 1). From the reference point t5, the curing time was reduced by a factor of two and four to define t5/2 and t5/4, respectively. The curing times determined in this way are summarized in Table 3. The ranking of the torque after t95 remains the same for both curing temperatures due to the modulus of the two different blends.

After curing, the compounds were immediately quenched with cold water to stop the curing process. The blend morphologies at the different curing times were compared to those observed after full conversion.

### 2.3. Characterization

#### 2.3.1. AFM

The influence of curing temperature and curing time is characterized by AFM experiments using the peak force quantitative nanomechanical mapping mode (PF-QNM™) from Bruker, Santa Barbara, CA, USA. The AFM mode PF-QNM offers the opportunity to perform quantitative analysis of mechanical properties in nanometer scale along with imaging of the surface topography. The probe was oscillated so that the probe and the sample were in intermittent contact with each other for a short period of time. In PF-QNM, the modulation is performed at a frequency of only 1 or 2 kHz, which is far below the resonance frequency of the cantilever in tapping mode [22]. At each tip-sample contact, force-separation curves were collected. These curves are used to calculate mechanical properties of the materials [23]. Hereby, the elastic modulus was determined using the Dejaguin–Muller–Toporov model [24]. The adhesion force between tip and sample was given by the minimum of the retrace curve. A nanomechanical study regarding the microstructure of NR/SBR blends has been published elsewhere [4].

For a smooth surface, the samples were cut with a cryo-ultramicrotome Leica EM UC6/EM FC6 (Leica Microsystems, Wetzlar, Germany) and equipped with a diamond knife. The cutting temperature was approximately 40 K below the glass-transition temperature of NR. Bulk sample with a flat surface was used for AFM investigations, while the thin sections cut from the surface were used for TEM. The investigations were performed on a Dimension Icon AFM with a NanoScopeV controller from Bruker. Measurements were carried out at ambient conditions in PF-QNM mode at a frequency of 1 kHz. A cantilever (RTESPA-150, Bruker) with a nominal spring constant of 6 N/m and a radius of 8 nm was used. For the morphology investigations of the blends, two different scanning areas of 5 × 5 µm^2^ and 10 × 10 µm^2^ were chosen with a resolution of 256 × 256 pixels.

#### 2.3.2. TEM

For TEM investigations, 60 nm thin sections were prepared by cryo-ultramicrotomy using a diamond knife with the same conditions as the AFM sample preparation mentioned above. The sections were investigated with a JEM-1400 (Jeol, Tokyo, Japan) at an acceleration voltage of 100 kV.

#### 2.3.3. Surface Free Energy

The wetting behavior between a liquid and a polymer can be used to derive the surface free energy of the polymer by measuring the contact angle *θ* of the two phases [25]. The calculation of the surface free energy can be done using Young’s equation (Equation (1)), in combination with the geometric mean model proposed by Owens and Wendt [26] (Equation (2)), where *γ_s_* is the surface energy of the solid, *γ_l_* the surface tension of the liquid, and *γ_sl_* the interfacial tension between the solid and the liquid.

(1) $$\gamma_{s}=\gamma_{sl}+\gamma_{l}\cdot \cos\theta$$

(2) $$\gamma_{sl}=\gamma_{s}+\gamma_{l}-2\sqrt{\gamma_{s}^{d}\gamma_{l}^{d}}-2\sqrt{\gamma_{s}^{p}\gamma_{l}^{p}}$$

The surface energy of the solid *γ_s_* and liquid *γ_l_* consists of two additive terms [27].

(3) $$\gamma =\gamma^{p}+\gamma^{d}$$

The first term in Equation (3) is the so-called polar part of the surface energy which describes the interaction between polar functional groups, and the second term is refers to van der Waals forces, and is the dispersive part of the total surface energy. Thereby, $\gamma_{l}^{p}$ and $\gamma_{l}^{d}$ are the polar and dispersive components of the liquid, and $\gamma_{s}^{p}$ and $\gamma_{s}^{d}$ are the polar and dispersive components of the solid, respectively.

Smooth surfaces are preferable to calculate the surface free energy of the polymer by contact angle measurements. For this reason, the raw elastomers are pressed between two smooth plates for 5 min at a temperature of 80 °C and a pressure of 4.5 bar (Labor Press P200S, Vogt, Berlin, Germany). The sample surface is protected from surface contamination by a foil until carrying out the contact measurements. The contact angle measurements were performed using the sessile drop method with a Mobile Surface Analyzer (Kruess, Hamburg, Germany). Two liquids were used to determine the polar and dispersive components of the polymer: distilled water (Fischer Scientific, Schwerte, Germany) and diiodomethane (Merck KgaA, Darmstadt, Germany, 98.5%). The investigations were performed at ambient conditions at a temperature of 22 °C.

#### 2.3.4. Broadband Dielectrically Spectroscopy (BDS)

The measurements were performed on a Concept 41 BDS system (Novocontrol Technologies GmbH, Montabaur, Germany) consisting of an Alpha-A High Performance Frequency Analyzer and a Novocool System for temperature control. The samples were mounted between two gold electrodes with a diameter of 30 mm. The measurements were carried out in a temperature range between −100 and 20 °C and in a frequency range between 0.1 and 2 × 10^7^ Hz.

## 3. Results and Discussion

### 3.1. AFM Visualization of NR/SBR Blend Morphology

In order to confirm the capabilities of AFM for visualizing domain morphologies of NR/SBR blends, the influence of the vinyl content of SBR on the morphology has been analyzed on the samples NR/SBR–LV and NR/SBR–HV introduced above. The AFM results were compared with standard methods, like TEM, DSC, and BDS. The expectation is that miscibility improves with increasing vinyl content [12,13,14]. Possible explanations for this behavior in the case of the specific elastomer blends investigated in this work are given at the end of this section.

**Figure 2:**
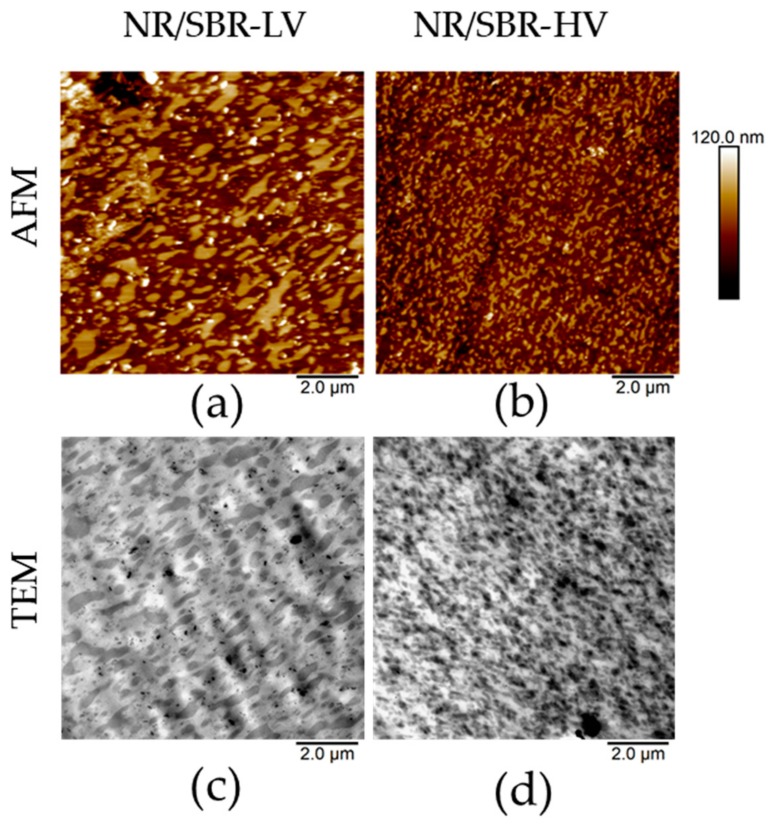
Comparison of atomic force microscopy (AFM) topography maps with TEM images: AFM topography maps of (**a**) NR/SBR–LV and (**b**) NR/SBR–HV and TEM images of (**c**) NR/SBR–LV and (**d**) NR/SBR–HV. The samples were cured at 160 °C.

Figure 2a,b show topography maps for the blends cured at a temperature of 160 °C. Both compounds reveal a continuous phase/discrete zone structure in which NR is the continuous matrix, and SBR forms the discrete zones. The SBR domains are sticking out of the NR matrix due to different tensions in the blend. Sectioning the blends allows for the release of tension and results in expansions and contractions of the phases in the blends [28], which creates the characteristic topographies shown in Figure 2a,b.

However, the morphologies created in the two blends are dissimilar, and show a clear dependency on their microstructures. The NR/SBR–HV blend exhibits much smaller domains with a narrower size distribution in comparison to the NR/SBR–LV blend. Consequently, the miscibility of SBR–HV in NR is improved. These investigations are consistent with literature results [12,13,14]. As expected, these observations show that AFM is an appropriate method to characterize the morphology and miscibility directly.

Nevertheless, the AFM results obtained with the samples mentioned above are compared to selected standard methods. On one hand, TEM is a direct visualization method, and on the other, DSC and BDS measurements are commonly used as indirect evaluation methods.

The observations made by AFM can be confirmed by TEM investigations. Figure 2c,d represent TEM results for the two blends with the same magnification as the AFM images above. TEM images reveal similar characteristics of the blend morphologies to AFM analyses; the size of SBR–LV domains is larger in comparison to that of the SBR–HV domains. Even the topological behavior in the AFM images is consistent with the TEM results. In unstained polymer blends, the mass–thickness contrast between different polymer phases is achieved through a variation in local thickness, since the densities are similar [29]. Thicker areas appear darker in the TEM images. In the case of the investigated blends, SBR is the darker phase, and thus, locally thicker than the NR phase.

**Figure 3:**
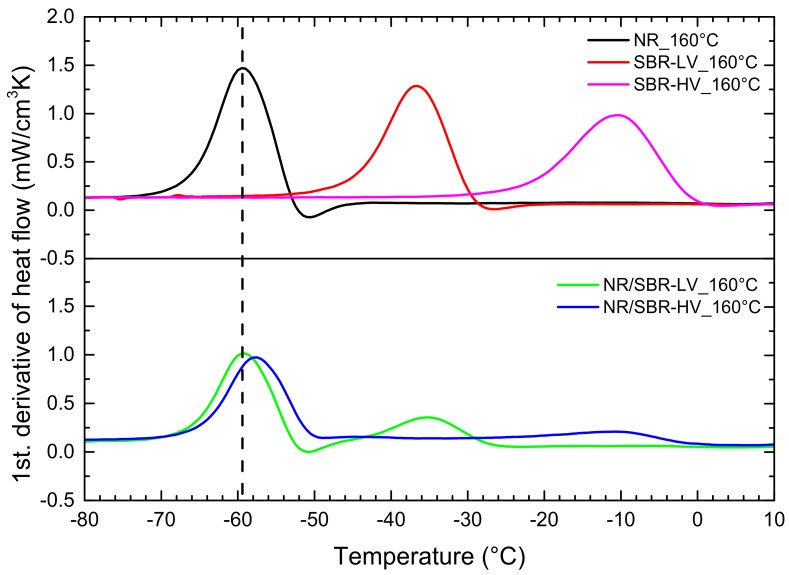
Differential scanning calorimetry (DSC) curves (first derivative) of the single NR, SBR–LV, SBR–HV, and their blends at a curing temperature of 160 °C.

DSC measurements were performed in order to compare a standard technique to the AFM results. Additionally to the blends, the DSC curves of the single elastomers were determined as reference samples. The results of the first derivative of the heat flow are shown in Figure 3. For the single elastomers, only one glass-transition peak (*T*_g_) was observed, whereas two glass-transition peaks were observed for the blends. Besides, small minima are visible for single NR, single SBR–LV, and their blends. These can be referred to as enthalpy relaxation [17,30,31]. The peaks of NR/SBR–LV are located at the same temperatures as the single phases of NR and SBR–LV, indicating immiscibility of these two components. In the case of NR/SBR–HV, a shift in *T*_g_ of NR to higher temperature is observed. Simultaneously, the intensity of the SBR–HV peak decreases, and in addition, a broadening of the peak is visible. The observation reveals a better miscibility of NR and SBR–HV. The DSC results confirm the findings of AFM. Nevertheless, from DSC measurements, it is only possible to get an indirect estimation of the miscibility, whereas AFM gives a direct impression.

**Figure 4:**
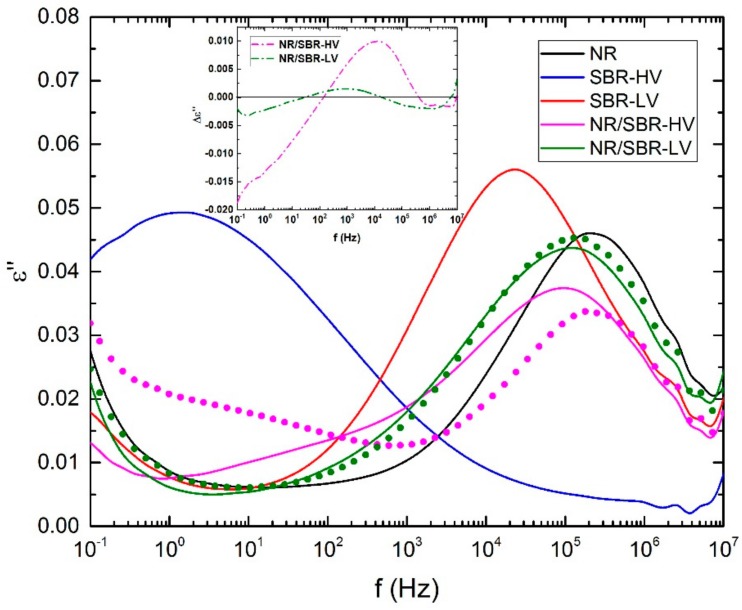
Imaginary permittivity data at 0 °C as a function of frequency for the single elastomers (NR, SBR–HV, and SBR–LV), and NR/SBR–HV and NR/SBR–LV blends. The circles represent the estimated dielectric loss using the weighted average of the individual components. The inset shows the different between the BDS measurements and the estimated values by the weighted average. The samples were cured at 160 °C.

The miscibility of the blend components can also be indirectly evaluated using broadband dielectric spectroscopy. Figure 4 shows the imaginary permittivity of the single elastomers and the blends at 0 °C as a function of frequency. The peak of the permittivity observed in Figure 4 for the different compounds corresponds to the α-relaxation of the material. Comparing the position of the α-peak for the single elastomers (NR, SBR–HV, and SBR–LV) indicates that the corresponding frequency of NR is the highest followed by SBR–LV and SBR–HV. This result is in agreement with the glass-transition measurements by DSC reported in Table 1. The α-relaxation peak in the case of NR/SBR–HV and NR/SBR–LV blends is mainly dominated by NR, due to the higher concentration. In order to estimate the degree of the phase separation between the two components of the blends, the dielectric loss of the blends is approximated by the weighted average of the raw elastomers (i.e., ${\varepsilon ”}_{blend}=0.7{\varepsilon ”}_{NR}+0.3{\varepsilon ”}_{SBR})$. This approach is common practice in BDS [32,33]. The resulting spectra are shown in Figure 4. As it can be seen, the estimated values of the dielectric loss using the weighted average in the case of NR/SBR–LV is very close to the measured data, indicating the formation of a completely phase-separated blend in this case (see the inset of Figure 4 for the difference between the measurement and the estimated data). In the case of NR/SBR–HV, however, the prediction of ${\varepsilon ”}_{blend}$ deviates from the actual measurements, signifying a partial miscibility with two phases enriched in one component. The improved miscibility observed with increasing vinyl content is in agreement with the AFM and TEM analysis (Figure 2).

The investigation of the specific blends in this work with the common approaches, TEM, DSC, and BDS, confirms the role of vinyl in increasing the miscibility of the elastomer combinations selected; this is in agreement with the AFM results.

### 3.2. State of Domain Morphology after Mixing

Before curing, the differences in domain morphology of the specific elastomer blends investigated in this work (NR/SBR–HV and NR/SBR–LV) can be explained from a different prospective. The Mooney viscosities of the blends are compared in Table 1. The viscosity of the raw elastomers differs less for NR and SBR–HV (96.2 to 68.0 Mooney units) compared with NR and SBR–LV (96.2 to 55.8 Mooney units). A larger difference in viscosity promotes the immiscibility of the polymers during mixing, since the break-down process reaches a different equilibrium state in balance with the coalescence [6]. These results are consistent with earlier studies [8,9,10,11]. 

The miscibility between two polymers can be described thermodynamically by the Gibbs free energy of mixing Δ*G* as the sum of enthalpic (Δ*H*) and entropic (Δ*S*) contributions (∆*G* = ∆*H* − *T*∆*S*, where *T* is temperature). For better miscibility, the free energy of mixing must be smaller [34]. The enthalpic contribution for an immiscible blend is positive due to unfavorable interactions between the polymers, and can be estimated from the difference in the solubility parameters (*δ*) of the polymers ($\Delta H\propto \Delta \delta^{2}$) [35]. Rocha et al. [36] measured the solubility parameters of NR and several SBR rubbers with different vinyl contents and constant styrene concentration. The results showed that the difference between the solubility parameters of NR and SBR decreases with increasing vinyl content. Based on these results, the enthalpy of mixing is expected to be smaller for a NR/SBR–HV blend compared to the NR/SBR–LV blend, and consequently, the miscibility improves with increasing SBR vinyl content, as shown in Figure 2. However, it is known that the solubility parameter is not an accurate approach to predict the miscibility of different polymers [37]. Therefore, the surface energy of the raw elastomers was determined and their contribution is discussed in the following.

The molecular origin of the difference between the enthalpy of mixing of NR/SBR–HV and NR/SBR–LV blends can be understood by comparing the dispersive and polar parts of the surface energy of the polymers. Table 4 summarizes the measured surface energies of the rubber used in this work, i.e., NR, SBR–HV, and SBR–LV. The results show that the dispersive part of the surface energy of SBR–HV and SBR–LV, which is mainly due to the van der Waals interactions between the polymer chains, is similar for the two polymers. However, the polar part of the surface energy of SBR increases with increasing vinyl concentration. Comparing NR and SBR surface energies indicates that SBR–HV has a comparable polarity to NR, therefore promoting the attractive interactions between NR and SBR–HV. Consequently, the effect of the repulsive interactions between the two components on the blend miscibility is reduced with increasing vinyl content. This explains the lower enthalpy of mixing of NR/SBR–HV compared to NR/SBR–LV, which was estimated by the solubility parameters. It is worth noticing that the surface energy measurements were performed at room temperature, while the mixing process was carried out at a higher temperature. However, we do not expect to attain any conclusions by comparing the surface energy in a relative manner, since the surface energy is determined by the chemistry of the polymers.

### 3.3. Effect of Curing Temperature on Morphology

The effect of curing temperature on the miscibility of the blends was investigated by comparing the phase morphology of the samples cured at 140 and 160 °C. Increasing the temperature from the mixing to the curing stage can influence the miscibility. Hasegawa et al. [38] observed a lower critical solution temperature (LCST) for polyisoprene/deuterated polybutadiene blends, indicating phase separation of the components upon heating. A similar trend has also been observed for blends of NR with high vinyl SBR or BR [37,39,40]. The observation of LCST has been attributed to the weakening of the specific interactions between the components and the increased contribution of the free volume effects with changing temperature [41]. Thus, phase separation might be promoted in the NR/SBR blends studied in this work during curing, due to the high temperature process. However, in elastomer compounds with an accelerator/sulfur system a competitive cross-link reaction takes place. Consequently, it is expected that phase separation can be hindered by cross-linking, since the coalescence of the dispersed phase is prevented.

**Figure 5:**
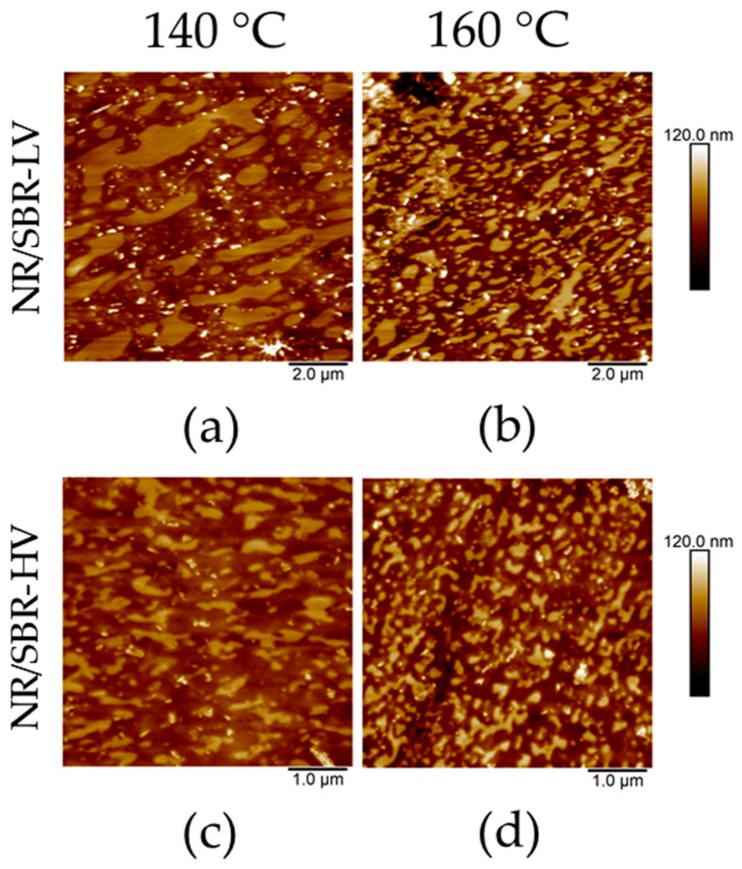
Comparison of topography images regarding the influence of curing temperature on blend morphology: NR/SBR–LV (**a**) at 140 °C and (**b**) at 160 °C; NR/SBR–HV (**c**) at 140 °C and (**d**) at 160 °C.

Figure 5 summarizes the morphologies formed at curing temperatures of 140 and 160 °C. Due to the smaller domain sizes of the SBR phase in SBR–HV blends, the corresponding scanning area is reduced (higher magnification) in the following images for this blend. 

At a curing temperature of 140 °C, NR/SBR–LV shows an increase in domain size compared to other compounds cured at 160 °C. Moreover, the size distribution becomes broad and the shape of the domains is distorted. This would lead to the conclusion that the cross-link reactions between the chains with a shorter scorch time at higher temperatures (see Figure 1) hinder phase separation upon heating. This is discussed in more detail in the next section where the phase morphologies are analyzed as a function of curing time. A similar influence of the temperature is observed for NR/SBR–HV; the effect is less pronounced for NR/SBR–LV. The higher sensitivity of the NR/SBR–LV blend to curing temperature is due to the less favorable enthalpic interactions between the components, as predicted by the surface energy measurements.

**Figure 6:**
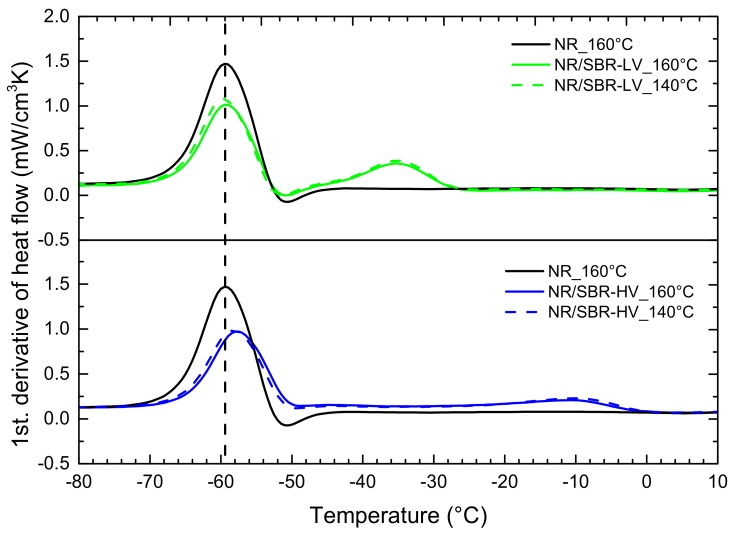
DSC curves (first derivative) of NR/SBR–LV and NR/SBR–HV at curing temperature of 140 and 160 °C.

In Figure 6 the DSC curves for the samples cured at 140 °C were compared to those cured at 160 °C. In both blends, the glass-transition temperatures of the individual elastomer phases move away from each other at a curing temperature of 140 °C in comparison to a curing temperature of 160 °C. This observation indicates a decrease in miscibility. Despite the vast changes in morphology in the AFM analysis for the two different temperatures in the NR/ SBR–LV blend, the shifts in *T*_g_ are quite small, indicating the limits of the DSC method and the potential of AFM.

### 3.4. Effect of Curing Time on Morphology

In diene–elastomer compounds, sulfur cross-linking is formed during curing. Therefore, phase separation for the LCST elastomer blends can be hindered at higher curing temperatures. Thus, the effect of curing time on blend morphology at different curing temperatures had to be investigated. Figure 1 shows the curing curves for the two types of blends discussed above at curing temperatures of 140 and 160 °C. As it can be seen, the scorch times at 160 °C are much shorter in comparison to those at 140 °C. In other words, the cross-links are created significantly faster upon increasing the temperature to 160 °C, and thus, the coalescence of dispersed domains can be hindered. However, due to longer scorch times during curing at 140 °C, the elastomers have much more time to be phase separated.

In order to confirm the assumption that a longer scorch time at 140 °C allows extended coalescence of the elastomers, the changes in morphology in the early stages of curing, defined by the onset point t5 (see Table 3), were analyzed by AFM. For a better visualization, the adhesion map of PF-QNM is used to characterize the growth in domain size. It has been shown that the adhesion maps reflect the morphology in the same appropriate way as the topography maps, but the results are less prone to artifacts, like knife marks, blooming, and other surface irregularities, which partly hinder the evaluation of the topography maps.

**Figure 7:**
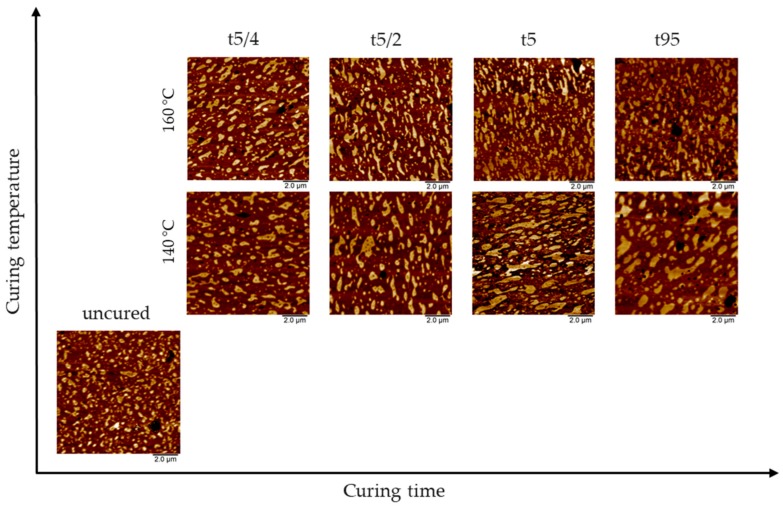
Comparison of adhesion maps regarding the influence of curing time and temperature on blend morphology in NR/SBR–LV blends. Curing times are defined according to Table 3.

Figure 7 illustrates the influence of curing time and temperature at the early curing stages, i.e., t5/4, t5/2, and t5, in comparison to the uncured compound and a fully cured NR/SBR–LV blend (t ≥ t95). Comparison of the uncured and fully cured states indicates that increasing temperature during curing leads to an increase in domain size as expected for a blend with a LCST. Nevertheless, the phase separation is higher at lower curing temperature. Consequentially, the other aspect to be considered is the hindering of the phase separation by the start of the cross-linking reaction.

At lower curing temperature and with proceeding curing time, the size of the domains increase, and their distribution becomes broader. Moreover, the domain structure varies from spherical to distort. The largest step in growth for the NR/SBR–LV blend at 140 °C was observed between the curing times of t5/2 = 13.5 min to t5 = 27 min, whereas no further increase in domain size is noticeable for the fully cured sample (47 min). This shows that if cross-linking is initiated, a further coalescence of the elastomers is not possible. For test samples cured at 160 °C, only a slight increase of the domain size is observed in comparison to the uncured compound. It seems that at short curing times, typical of high temperatures, a certain degree of cross-linking is hinders the coalescence of the elastomers. These findings can be explained by the curing curves in Figure 1. The vulcanization at 160 °C leads to an immediate start of the conversion. In contrast, the curing curve for the vulcanization at 140 °C shows a long scorch time (about 27 min) before the cross-link reaction starts. In this period, the elastomers have time for coalescence.

Analogous observations were made by Yamanaka et al. [42] in rubber-modified epoxy resin. As the reaction progressed, phase separation appeared, but was slowed down by competitive network formation. At higher temperature, this separation progressed faster, but it was also hindered quite early due to cross-linking.

**Figure 8:**
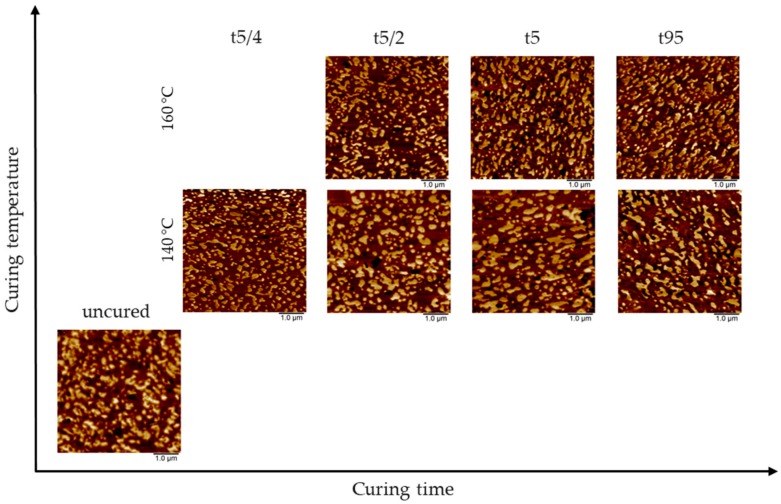
Comparison of adhesion maps regarding the influence of curing time and temperature on blend morphology in NR/SBR–HV blends. Curing times are defined according to Table 3.

For the NR/SBR–HV blend, a similar behavior of the morphology was evident; the results are shown in Figure 8. At 160 °C, only negligible differences were noticeable with changes in curing time. Otherwise, slightly larger areas of the NR matrix were visible at the lower curing temperature (140 °C). In summary, the influence of the curing temperature is less pronounced for the more miscible blend, and therefore, only smaller changes in morphology can be observed. This behavior can be explained by considering the role of the surface free energy. NR and SBR–HV have similar values for the polar parts of the surface energy, and thus, these elastomers have only a small tendency for phase separation during coalescence time, although the scorching time is quite long and similar to NR/SBR–LV. These results show that the tendency for phase separation is more pronounced for elastomers with larger differences in the polar part of the surface free energy.

## 4. Conclusions

In this paper, AFM measurements were performed on NR/SBR blends with differing vinyl content to investigate the influence of curing temperature and curing time on their blend morphology. In addition, the well-known influence of vinyl content on miscibility was confirmed by AFM for these specific blends. The AFM results showed a smaller domain size for the blend with the higher vinyl amount in the SBR. The decrease in domain size with increasing vinyl content indicates an improved miscibility between NR and SBR–HV. These results are consistent with the ones obtained with standard techniques, like DSC, TEM, and BDS.

The effect of vinyl content on phase morphology is discussed by comparing the polar part of the surface free energy, solubility, and viscosity. An increase in vinyl concentration of SBR leads to an increase in the polar part of the surface free energy to a value closer to the one of NR. This leads to an increase in the attractive interactions between the components, suppressing the effect of the unfavorable enthalpic interactions. 

Heating the samples during curing leads to an increase in domain size for both types of blends in comparison to the uncured state. These observations are consistent with the described LCST behavior of NR/SBR blends in the literature, where phase separation is predicted upon heating [37,39,40]. Nevertheless, the increase in domain size is much larger for curing temperatures of 140 °C compared to 160 °C. This seemingly contradictory behavior is due to the faster cross-linking at higher temperatures and the subsequent hindering of the coalescence. It is remarkable that the increase in domain size is much larger for the poorly miscible NR/SBR–LV blend. The difference in polarity between NR and SBR–LV is the driving force for phase separation to minimize the surface contact.

## Figures and Tables

**Table 1:** Characteristics of the pure polymers.

Properties	NR	SBR–LV	SBR–HV
Styrene/vinyl content (%)	-	24/34.2	24.3/67.2
*T*_g_ (DSC) ^a^ (°C)	−60	−37	−12
Mooney viscosity ^b^ [ML1 + 4 (100 °C)]	96.2	55.8	68.0

^a^ DSC measurements were carried out at a heating rate of 10 K/min in a temperature range from −150 to 70 °C using a DSC1 (Mettler-Toledo, Greifensee, Switzerland). ^b^ Mooney Viscosity was determined with a MV2000 (Alpha Technologies, Hudson, OH, USA).

**Table 2:** Compound recipes in phr (parts per hundred rubber).

Ingredients	NR/SBR–LV	NR/SBR–HV
NR	70	70
SBR–LV	30	-
SBR–HV	-	30
6PPD ^1^	1	1
Zinc oxide	2	2
Steric acid	1	1
TBBS ^2^	2	2
Sulfur	2	2

^1^ N-alkyl-N’-aryl-p-phenylenediamine. ^2^ N-tert-Butylbenzothiazol-2-sulphenamide.

**Table 3:** Overview of curing times at the two curing temperatures. Curing times indicate the conversion level: t95 defines the time for 95% conversion and t5 refers to the time for 5% conversion.

	Curing Time (min)			
Conversion Time	NR/SBR–LV 140 °C	NR/SBR–LV 160 °C	NR/SBR–HV 140 °C	NR/SBR–HV 160 °C
Uncured	0	0	0	0
t95	47	13.8	47	12.8
t5	27	7.7	26	6.7
t5/2	13.5	3.9	13	3.4
t5/4	6.8	1.9	6.5	-

**Table 4:** Surface free energy of the raw elastomers.

Surface Free Energy (mN/m)	NR	SBR–HV	SBR–LV
Dispersive part	20.2	30.8	29.9
Polar part	5.5	4.0	1.6
